# Effect of Hemicellulose on the Wet Tensile Strength of *Kozo* Paper

**DOI:** 10.3390/molecules28196996

**Published:** 2023-10-09

**Authors:** Zhiyou Han, Keiko Kida, Kyoko Saito Katsumata, Masaki Handa, Masamitsu Inaba

**Affiliations:** 1 Conservation Standards Research Institute, The Palace Museum, Beijing 100009, China; 2 Conservation Science Laboratory, Graduate School of Conservation, Tokyo University of the Arts, Tokyo 110-8714, Japan; kida.keiko@fa.geidai.ac.jp (K.K.); katsumata.kyoko@fa.geidai.ac.jp (K.S.K.); 3 Graduate School of Agricultural and Life Sciences, Faculty of Agriculture, University of Tokyo, Tokyo 113-8654, Japan; 4 Handa Kyuseido Co., Ltd., Tokyo 151-0064, Japan; masaki-handa@kyuseido.com

**Keywords:** paper-based cultural heritage, wet tensile strength, Japanese paper, xylan, hemicellulose, mounting paper, analysis techniques

## Abstract

*Kozo* paper, *usu-mino-gami*, is frequently used as the first back lining paper of hanging scrolls in order to support the main paper with a painting or a work of calligraphy on it. To dye it an appropriate color, paper is often treated with an alkali mordant solution. However, current *kozo* paper products have received such comments from conservators that wet tensile strength is weak and hard to handle. Therefore, improving the wet tensile strength of *kozo* paper is required. In previous papers, the effect of the sheet forming method, cooking condition, and parenchyma cell content between fibers on the wet tensile strength of *kozo* paper has been investigated. In this paper, the effect of glucuronoxylan, the main component of hardwood hemicellulose on the wet tensile strength of *kozo* paper was investigated. The wet tensile strength of *kozo* paper, when made in different cooking conditions, was evaluated using the Finch device. Glucuronoxylan content in fiber was analyzed using GC-FID. According to the results, it has been proved that glucuronoxylan content (with a xylan to glucan molar ratio of 4.43% to 5.16%) itself contributes to the wet tensile strength of the *kozo* sheet. Therefore, to increase the wet tensile strength of *kozo* paper, it is recommended to cook under milder conditions, thus retaining a higher amount of glucuronoxylan in the pulp.

## 1. Introduction

In Japanese mounted paintings, a paper-based backing is used to provide direct support for the delicate main paper. This backing sheet, called “*hada-ura-gami*”, is especially used with hanging scrolls, which commonly employ a thin grade of “*usu-mino-gami*” (Japanese *kozo* paper) as their primary lining. This first backing paper is often dyed to achieve a better tone and color of the image on the main paper and is adhered during mounting; therefore, a high wet tensile strength is essential.

*Usu-mino-gami*, made by the late Japanese papermaker Kozo Furuta, gained renown for its impressive wet strength. The paper made by his apprentice, Satoshi Hasegawa, was comparatively weak, leading Hasegawa to seek out the reason for this disparity. The resulting research lays the foundation for the present study.

In the initial study [1], we analyzed sheet samples that had been prepared under various cooking conditions by different sheet formers and produced using different sheet-forming techniques. The wet tensile strength of these samples was then assessed using the Finch method. The results show that wet tensile strength became higher with milder cooking conditions, such as a shorter cooking time and mild alkali cooking agent.

In the subsequent investigation [2], we explored the relationship between wet tensile strength and parenchyma cell content (defined as the proportion of the film area located between fibers on the surface area of the sheet). Our examination encompassed *kozo* sheets that had been prepared with identical alkali concentrations (of either sodium carbonate or sodium hydroxide) but different cooking times, as well as *kozo* sheets produced using the same cooking durations but with different alkali concentrations. Additionally, we evaluated *kozo* sheets made while maintaining the same cooking conditions but gradually reducing the parenchyma cell content using a fiber classier (screen opening 75 μm). Our findings revealed a direct correlation between higher wet tensile strength and increased parenchymal cell content.

The main chemical components of *kozo* paper are cellulose, hemicellulose, lignin, and pectin. According to Ishii [3], the xylem of hardwood typically contains approximately 20–30% hemicellulose, of which 80–90% is *O*-acetyl-4-*O*-methylglucuronoxylan (also known as glucuronoxylan). This is followed by glucomannan, the next most abundant hemicellulose found in hardwood. Since *kozo* paper originates from ligneous bark, it is likely that its composition is similar to the aforementioned hemicelluloses.

Some of the hemicellulose in wood becomes degraded and is lost during the cooking process. This occurs because of the peeling reaction, a process in which the 1→4-linked polysaccharides of cellulose and hemicellulose are successively removed from their reducing end groups during alkali treatment. As a result, both compounds undergo a reduction in their degree of polymerization. Because the original degree of polymerization for cellulose is relatively high, the effect of depolymerization is small, although their yield is reduced. In the case of glucomannan, however, the original degree of polymerization is around 70, so this peeling reaction contributes significantly to a reduction in the degree of polymerization [4]. Consequently, relatively significant amounts of glucomannan are lost during cooking due to its low molecular weight [5]. Glucuronoxylan, by contrast, exhibits a higher resistance to degradation at temperatures below 140 °C. The peeling reaction stops at the residue with 4-*O*-methyl glucuronic acid at the C-2 position and acetyl groups at the C-2 and C-3 positions [6]. Danielsson [7] reported that glucuronoxylan is reabsorbed on the fiber when the alkali concentration decreases during the late stages of cooking, as well as when the temperature decreases. Danielsson and Lindstom [5] reported that when glucuronoxylan from birch wood is adsorbed onto softwood pulp, the resulting increase in the higher degree of polymerization as well as more carboxyl group densities of glucuronoxylan leads to greater adsorption onto the fiber surface. Additionally, Mitikka-Eklund et al. [8] confirmed that glucuronoxylan was absorbed on the outer surface of the fiber.

Schonberg et al. [9] reported that the addition of glucuronoxylan and its adsorption onto the fibrous surface increased tensile strength. However, they noted that the glucuronoxylan retained within the fiber did not affect tensile strength or tear strength. Ban et al. [10] stated that the damage to cellulose and hemicellulose caused by cooking has a greater impact on paper quality than the relationship between the tensile strength of the sheet and the fiber’s hemicellulose content.

Robinson reviewed the effect of hemicellulose on fiber bonding as follows. Since hemicellulose is flexible, it provides more hydrogen bonds per unit area than the cellulose surface. Furthermore, hemicellulose has a molecular chain with small molecular weight and few steric obstructions; therefore, the hydroxyl groups of hemicellulose can easily bind with hydroxyl groups on the surface of cellulose, which lacks mobility [11].

In particular, glucuronoxylan has a side chain containing a carboxyl glucuronic acid, which gives rise to a stronger hydrogen bond with hydroxyl groups such as cellulose. As mentioned above, the amount of glucuronoxylan content, the main component of hardwood hemicellulose, is known to contribute to paper strength.

Furthermore, in the papermaking process, because fiber itself is relatively rough and elastic, it must be beaten before becoming pulp. The fiber bundles are broken up to present fibrillation. Fibers also transfer to the appropriate length, and the surface also becomes gelatinized via a beating process. They expand and become plastic when exposed to water. Because hemicellulose has a strong hydrophilicity, when it is present on the surface of the fiber, water causes it to swell. The overall plasticity of the fiber is thus enhanced and becomes gelatinized more easily. When making paper, the strength of the paper increases due to the increase in the combination of strong plastic fibers [12].

In this paper, we began by examining glucuronoxylan content in the fibers of the *usu-mino-gami* made by Hasegawa. After this, we analyzed samples produced using different cooking methods in light of the findings on wet tensile strength gleaned from our earlier studies. We then investigated glucuronoxylan content following its extraction from *kozo* chips and its addition to *kozo* pulp when treated under identical cooking conditions and adsorbed onto the fibers. The purpose of this study was to clarify the effect of glucuronoxylan on the wet tensile strength of paper. It provides a guideline for the production of wet tensile strength *kozo* paper and contributes to the preservation and conservation of cultural properties with good paper quality.

## 2. Result and Discussion

### 2.1. Usu-Mino-Gami

Neutral carbohydrate components in Hasegawa-made *usu-mino-gami* were analyzed, with results normalized by molar ratio to glucan content (Table 1). Here, all the xylan was derived from glucuronoxylan, and the mannan was derived from glucomannan. Therefore, the neutral carbohydrate value indicates the relative amount of these hemicelluloses. Notably, the *usu-mino-gami* made by Furuta (Fu) exhibited not only a high glucuronoxylan content but also a high glucomannan content, indicating milder cooking conditions.

**Figure 1:**
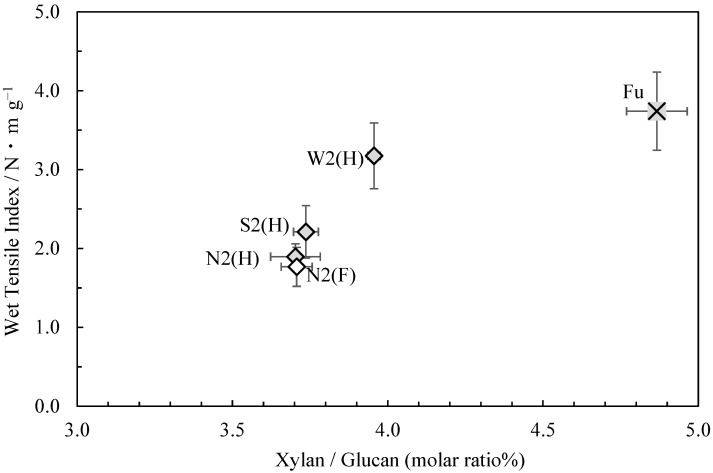
The relationship between xylan/glucan molar ratio and the wet tensile index for *usu-mino-gami*.

Figure 1 shows the relationship between the wet tensile index (in 44 mmol/L potassium hydroxide solution) [1] and glucuronoxylan content (the molar ratio of xylan to glucan) of the *usu-mino-gami* reported in the first paper. As can be seen, a higher wet tensile index correlated with a higher glucuronoxylan content. Consistent with this finding, the sample with the highest wet tensile index, Fu, also had the highest glucuronoxylan content, while the sample with the lowest wet tensile index, N2(F), had the lowest glucuronoxylan content. These results suggest that weaker cooking conditions are conducive to enhancing the wet tensile strength of *usu-mino-gami* and that glucuronoxylan content increases in the process.

However, interpreting the results related to the Hasegawa sample proved challenging due to varying cooking conditions, specifically due to changes in the alkali concentrations and cooking time. Therefore, we had *kozo* chips from Kochi Prefecture and manufactured *kozo* sheets with the same alkali concentration or a constant cooking time. The experimental results for *kozo* sheets are shown below.

### 2.2. Kozo Sheets Produced with Identical Alkali Concentrations and Different Cooking Time

The results of the neutral carbohydrate analysis of *kozo* sheets prepared using identical alkali concentrations with different cooking durations are shown in Table 2 and Table 3. The neutral carbohydrate content derived from all hemicelluloses was higher in the sample cooked in sodium carbonate (KC) than in the sample cooked in sodium hydroxide (KH) when the same cooking time was used. This finding aligns with previous research by Gustavsson, AL-Dajani [13], and Jiang et al. [14], who reported that in wood pulp, elevated alkalinity causes hemicellulose to degrade and dissolve more easily. Therefore, it comes as no surprise that the hemicellulose content in the fiber of samples cooked with sodium hydroxide (KH), which is highly alkaline, is notably diminished compared with that which was cooked with sodium carbonate (KC).

**Figure 2:**
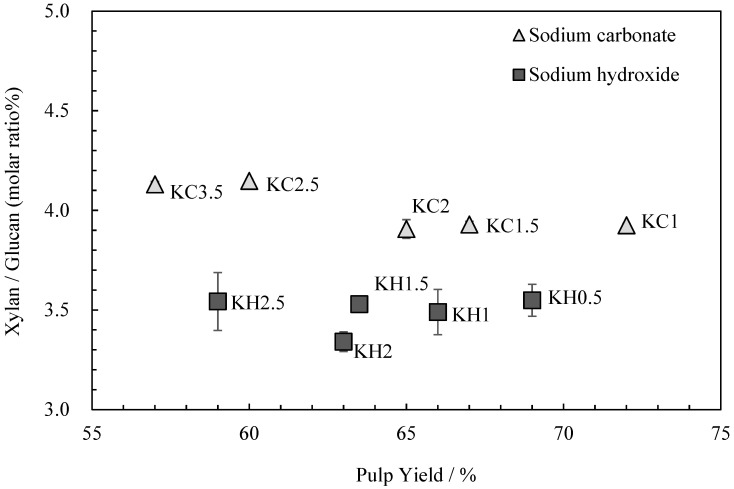
The relationship between xylan/glucan molar ratio and the pulp yield for *kozo* sheets prepared using different cooking agents and times.

The relationship between the *kozo* sheets’ glucuronoxylan content and pulp yield is shown in Figure 2. Although Gustavsson, AL-Dajani [13] and Jiang et al. [14] reported a decrease in glucuronoxylan content with longer cooking times, Clayton and Stone [15] stated that since glucuronoxylan is reabsorbed following cooking, cooking times have little impact on the final content. In this study, the glucuronoxylan content was almost the same with each alkali, regardless of the cooking time.

**Figure 3:**
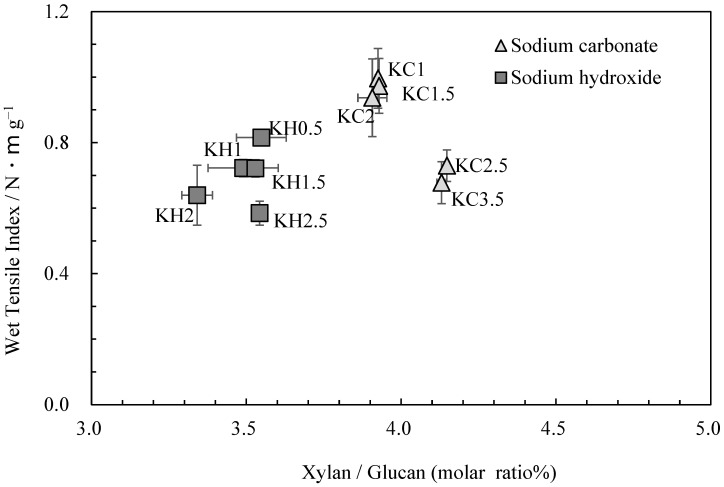
The relationship between xylan/glucan molar ratio and wet tensile index for *kozo* sheets.

Figure 3 shows the relationship between the glucuronoxylan content and wet tensile index. While the wet tensile index was found to decrease as cooking time increased, glucuronoxylan content changed little when the same cooking agent was employed. This finding underscores the role of other factors, such as parenchyma cell content and the degree of cellulose damage, in influencing changes in strength [2]. However, it should be noted that the amount of glucuronoxylan content varied when different cooking agents were used, directly impacting wet tensile strength.

### 2.3. Kozo Sheets Produced with Uniform Cooking Times but Different Alkali Concentration

**Figure 4:**
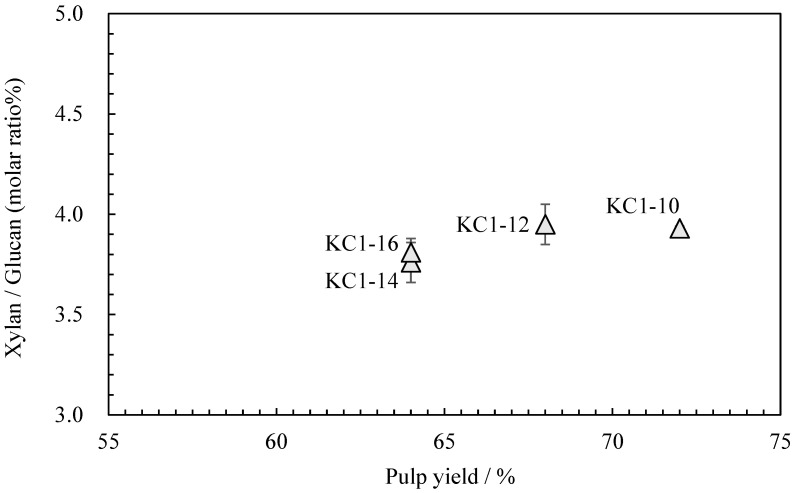
The relationship between pulp yield and xylan/glucan molar ratio of sodium carbonate when cooking *kozo*.

**Figure 5:**
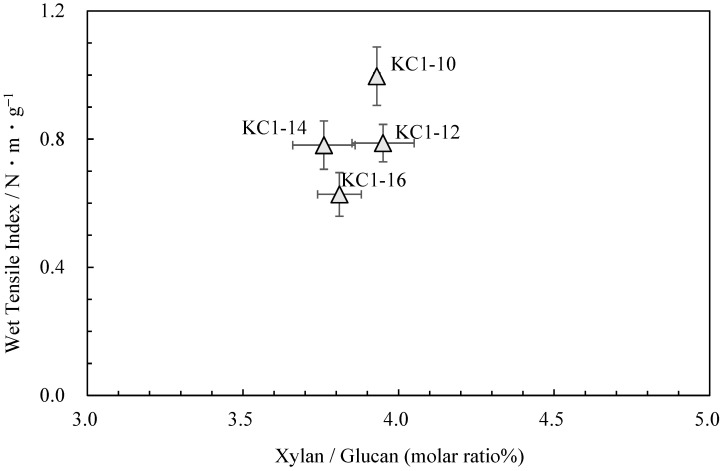
The relationship between xylan/glucan molar ratio and wet tensile index for *kozo* sheets of sodium carbonate when cooking.

Neutral carbohydrate analysis was performed on the *kozo* sheets by varying the sodium carbonate concentration while maintaining the same cooking time (Table 4). Figure 4 shows the relationship between the pulp yield and glucuronoxylan content. Among KC1-10 (KC1 in the previous chapter), KC1-12, KC1-14, and KC1-16, the glucuronoxylan content tended to decrease as the alkali concentration increased. Figure 5 shows the relationship between these data and the wet tensile index [2], where it can be observed that a higher wet tensile index is correlated with lower alkali concentrations. Additionally, wet tensile strength is linked to the amount of glucuronoxylan content.

### 2.4. Kozo Sheets Made with Absorbing Glucuronoxylan

The above experiments indicate a correlation between the glucuronoxylan content and the wet tensile index. However, different cooking conditions not only affect the content of hemicellulose but also cause varying degrees of damage to cellulose and parenchyma cell content, which probably also leads to an effect on paper strength. Therefore, in the following paragraph, in order to explore the relationship between glucuronoxylan content and wet tensile strength, cooking conditions were unified to ensure that glucuronoxylan content was the only variable.

**Figure 6:**
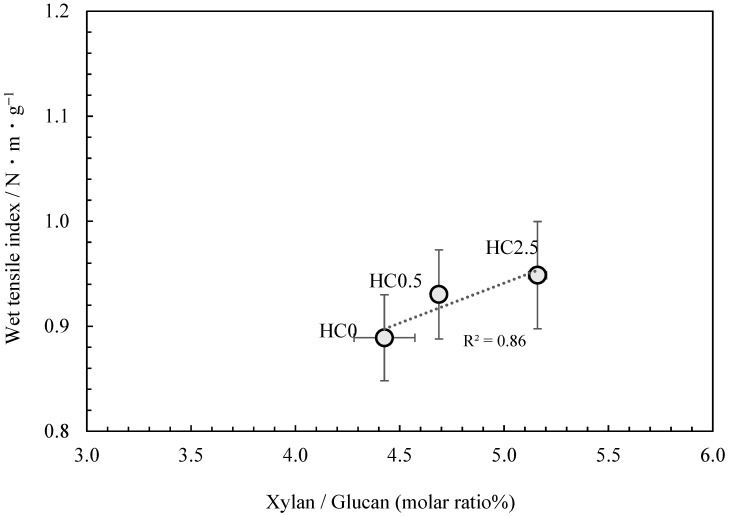
The relationship between xylan/glucan molar ratio and the wet tensile index for *kozo* sheets when xylan content is added.

Therefore, a strategy was employed in which glucuronoxylan extracted from *kozo* chips was added to *kozo* pulp that had been cooked under identical conditions. The glucuronoxylan content was then adsorbed onto the fibers, resulting in *kozo* paper samples that differed only in their glucuronoxylan content, allowing for its specific effect on paper strength to be investigated. The results of the neutral carbohydrate analysis are shown in Table 5. Though glucuronoxylan content was added just before cooking concluded, the proportion measured in the samples only ranged from 4.43% to a maximum of 5.16% for the xylan to glucan molar ratio. Figure 6 shows that the addition of glucuronoxylan content increased the wet tensile index, and although this phenomenon is known to occur in the production of wood pulp-based paper, our results confirm that a similar effect takes place when glucuronoxylan content is adsorbed onto the fibers of *kozo* paper.

## 3. Experimental Section

### 3.1. Sample

#### 3.1.1. Hasegawa’s *Usu-Mino-Gami*

Five samples were prepared at Hasegawa Washi Kobo, Satoshi Hasegawa’s paper mill (Table 6). Nasu *kozo* white bark was cooked using sodium carbonate (Na_2_CO_3_) to form a sheet. Sample N2 represents a specimen made by Hasegawa according to his usual cooking process and conventional papermaking techniques. For sample N2(F), the same stock was bag-washed to remove parenchyma cells and fine fibers. The creation of sample S2 entailed an increase in cooking agent concentration and a shorter cooking time, while for sample W2, the cooking agent concentration was decreased, but the cooking time was likewise short. The final sample, Fu, was made by the late Furuta [1].

#### 3.1.2. Kozo Sheets Produced with Identical Alkali Concentrations or Uniform Cooking Time

For the preparation of *usu-mino-gami*, the alkali concentration and cooking time were adjusted to vary the cooking strength. As shown in Table 7, these *kozo* sheets were prepared according to the Tappi standard handmade method (JIS P8222:2005) [17] with a grammage of around 30 g/m^2^ [2].

#### 3.1.3. Samples with Different Glucuronoxylan Content under Identical Cooking Conditions

Glucuronoxylan was isolated according to the following procedure. Kozo chips were extracted with methanol in a Soxhlet extractor for 6 h. The air-dried chips were then stirred in 4 L of distilled water for 2 days at room temperature to extract the water-soluble components. After additional air-drying, glucuronoxylan was extracted using 1 L of a 10% KOH solution for 3 h at room temperature under a nitrogen flow with agitation. Following suction filtration using ADVANCTEC No. 4A filter paper, the filtrate was introduced into 2.6 L of methanol containing 0.125 L acetic acid while stirring. The precipitated glucuronoxylan was collected via centrifugation and washed sequentially with 80% ethanol, anhydrous ethanol, and ethyl ether. The resulting crude glucuronoxylan was then re-dissolved in 1 L of a 5% KOH solution, purified via precipitation with 0.75 L ethanol, and the precipitate was again collected using centrifugation. It was then washed with ethanol and dried over phosphorus pentoxide under a vacuum in a desiccator using a rotary pump [18].

The following process was then used to add the glucuronoxylan to the pulp. First, pulp from Kochi *kozo* chips (60 g, cut into 4 mm lengths) was cooked in 1.8 L of 10% sodium carbonate solution for 50 min. Once it had cooled, the pulp was suction-filtered to separate the fiber from the cooking liquid. It was then divided into three equal parts according to weight. The cooking liquid was heated while nitrogen gas was blown into it. and when its boiling point was reached, glucuronoxylan was added, respectively, in amounts of 0.5 g and 2.5 g. These samples were named HC0.5 and HC2.5. The pulp was introduced and heated for 10 min, after which it was removed without cooling. The sample without the addition of glucuronoxylan underwent the same procedure (HC0). The pulp was washed as usual, and then it was beaten to 5000 revolutions in a PFI mill. *Kozo* sheets of gramarge at approximately 30 g/m^2^ were formed, as shown in Table 8 (JIS P8222:2005) [2].

### 3.2. Measurement of Wet Tensile Strength by Finch Method

**Figure 7:**
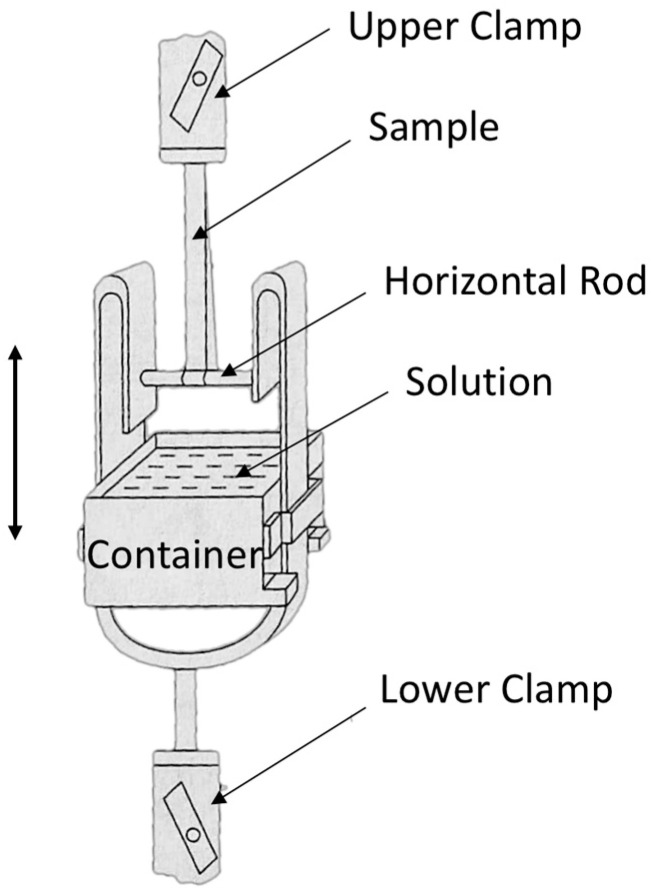
Finch device for short time wet strength measurement of paper (JIS P8135:1998).

Consistent with our initial report [1], the wet tensile index was assessed using the Finch method, which involves immersion in a 44 mmol/L potassium carbonate solution for 20 s (JIS P8135:1998) [19]. In the Finch device, a sample piece turns around a horizontal rod, and its two ends are held by an upper clamp. A water vessel (container) can move up or down. By lifting up the container, a part of the sample piece around the horizontal rod is immersed in the solution and kept for a scheduled time. Tensile testing can be performed immediately after lowering the container, as in Figure 7. Ten pieces were measured for each sample, and the means and standard deviations of the measurement results have been used in this paper.

Wet tensile strength is expressed below:

$$S_{w}=0.5\times \frac{X}{W\times n}$$

*S_w_*: Wet tensile strength (kN/m)

*X*: Maximum load until raptured (N)

*W*: Width of a sample piece (mm)

*n*: Number of layered sample pieces (*n* = 1 in our case)

Wet tensile strength when determined was divided on the basis of the weight of the sheet to obtain the wet tensile index.

### 3.3. Neutral Carbohydrate Analysis

Paper samples were disintegrated, and 50 mg of the oven-dried sample was weighed and subjected to sulfuric acid hydrolysis to form a monosaccharide. This was further reduced to alditol before being acetylated and analyzed using GC–FID [18]. The GC analysis conditions were as follows: Agilent 6890N; injection volume: 2 µL; column: Agilent J&W DB-225 (250 μm i.d., 30 m length, and 0.25 μm film thickness); oven temperature: 220 °C; analysis time: 30 min; inlet temperature: 250 °C; split ratio: 30:1; carrier gas: N_2_, 0.7 mL/min; detector: FID (Flame Ionization Detector).

In this study, inositol was used as an internal standard; however, since acid hydrolysis is not sufficient, it is not expressed as an absolute amount but as a ratio of glucan. Two to three points for each sample were taken, and the entire process was performed. Measurement results are shown as the mean and standard deviation.

## 4. Conclusions

Although the amount of glucuronoxylan content in the *kozo* sheets varied with different alkali concentrations and types, its content remained quite consistent under conditions in which the type of alkali used and the alkali concentrations were identical.

Consequently, variables affecting the wet tensile index include the alkali used, the cooking intensity with sodium hydroxide or sodium carbonate, and the concentration of sodium carbonate assessed with consistent cooking times. All of these factors were found to impact the amount of glucuronoxylan content in *kozo* sheets, contributing to differences in their wet tensile strength.

With regard to the samples that had an addition of glucuronoxylan extracted from *kozo* chips to *kozo* pulp, it was confirmed that the wet tensile strength of the *kozo* sheet increased in proportion to the amount of glucuronoxylan that was added. Since the degree of cellulose damage and parenchyma cell content in these sheets was kept constant, it could finally be reliably determined that the glucuronoxylan content itself was the factor contributing to the wet tensile strength of *kozo* paper. Therefore, to increase *kozo* paper’s wet tensile strength, sodium carbonate should be employed, and the fiber should be cooked under mild conditions, thus retaining a higher degree of glucuronoxylan content in the pulp. The cooking conditions that allow for the retention of a higher glucuronoxylan content are something to consider exploring in a future study.

The results of this paper intend to disclose information about the production of paper producers in the country. This allows conservators and producers to exchange information on issues such as defects in the use of the paper. We are convinced that the multifaceted discussions among producers, conservators, and researchers are based on objective data on the correlation between the raw materials and the manufacturing process of paper. The usability and stability of this paper provide significant support for the stable production of mounting paper.

## Figures and Tables

**Table 1:** Neutral carbohydrate composition of *usu-mino-gami* (molar ratio to glucan).

Molar Ratio to Glucan/%	Fu	W2(H)	S2(H)	N2(H)	N2(F)
Rhamnan	1.43 ± 0.01	1.34 ± 0.04	1.39 ± 0.02	1.05 ± 0.03	1.00 ± 0.01
Arabinan	0.54 ± 0.06	0.38 ± 0.02	0.43 ± 0.09	0.20 ± 0.04	0.24 ± 0.01
Xylan	4.87 ± 0.40	3.96 ± 0.01	3.74 ± 0.04	3.70 ± 0.08	3.70 ± 0.05
Mannan	1.76 ± 0.09	1.13 ± 0.10	1.08 ± 0.02	0.80 ± 0.02	0.61 ± 0.05
Galactan	1.87 ± 0.12	1.74 ± 0.03	2.15 ± 0.06	1.41 ± 0.01	1.57 ± 0.03
Yield of neutral carbohydrate */%	80.0 ± 0.6	82.7 ± 0.3	85.3 ± 0.4	83.8 ± 0.5	82.4 ± 0.6

* Including glucan yield.

**Table 2:** Neutral carbohydrate composition of *kozo* sheets using sodium carbonate to cook *kozo* fiber.

Molar Ratio to Glucan/%	KC1	KC1.5	KC2	KC2.5	KC3.5
Rhamnan	1.18 ± 0.02	1.12 ± 0.01	1.14 ± 0.03	1.01 ± 0.02	1.02 ± 0.03
Arabinan	0.23 ± 0.06	0.23 ± 0.01	0.26 ± 0.02	0.22 ± 0.00	0.23 ± 0.00
Xylan	3.93 ± 0.01	3.93 ± 0.02	3.91 ± 0.05	4.15 ± 0.01	4.13 ± 0.08
Mannan	1.12 ± 0.03	1.04 ± 0.03	0.94 ± 0.02	0.95 ± 0.07	0.85 ± 0.02
Galactan	1.56 ± 0.03	1.54 ± 0.01	1.48 ± 0.00	1.40 ± 0.01	1.42 ± 0.01
Yield of neutral carbohydrate */%	81.5 ± 0.6	81.9 ± 0.1	81.5 ± 0.2	79.9 ± 0.2	82.8 ± 0.1

* Including glucan yield.

**Table 3:** Neutral carbohydrate composition of *kozo* sheets using caustic sodium hydroxide to cook *kozo* fiber.

Molar Ratio to Glucan/%	KH0.5	KH1	KH1.5	KH2	KH2.5
Rhamnan	0.80 ± 0.03	0.71 ± 0.01	0.71 ± 0.01	0.69 ± 0.00	0.70 ± 0.01
Arabinan	0.10 ± 0.01	0.10 ± 0.02	0.09 ± 0.01	0.08 ± 0.00	0.12 ± 0.04
Xylan	3.55 ± 0.08	3.49 ± 0.11	3.53 ± 0.02	3.34 ± 0.01	3.54 ± 0.15
Mannan	0.61 ± 0.01	0.43 ± 0.01	0.34 ± 0.02	0.34 ± 0.03	0.33 ± 0.04
Galactan	0.85 ± 0.01	0.65 ± 0.00	0.53 ± 0.00	0.48 ± 0.01	0.56 ± 0.02
Yield of neutral carbohydrate */%	80.2 ± 0.3	77.8 ± 0.5	81.0 ± 0.2	83.0 ± 0.2	78.6 ± 0.9

* Including glucan yield.

**Table 4:** Neutral carbohydrate composition of *kozo* sheets prepared using different sodium carbonate concentrations to cook *kozo* fiber.

Molar Ratio to Glucan/%	KC1-10 *^1^	KC1-12	KC1-14	KC1-16
Rhamnan	1.18 ± 0.02	1.21 ± 0.08	1.19 ± 0.04	1.17 ± 0.06
Arabinan	0.23 ± 0.06	0.27 ± 0.01	0.18 ± 0.01	0.20 ± 0.01
Xylan	3.93 ± 0.01	3.95 ± 0.10	3.76 ± 0.10	3.81 ± 0.07
Mannan	1.12 ± 0.03	1.12 ± 0.05	1.05 ± 0.04	1.00 ± 0.10
Galactan	1.56 ± 0.03	1.59 ± 0.03	1.42 ± 0.02	1.42 ± 0.10
Yield of neutral carbohydrate *^2^/%	81.5 ± 0.6	92.3 ± 0.2	93.4 ± 0.2	92.9 ± 0.3

*^1^ Sample KC1-10 is the same sample as KC1 in the previous chapter. *^2^ Including glucan yield.

**Table 5:** Neutral carbohydrate composition of *kozo* sheets made with absorbing glucuronoxylan.

Molar Ratio to Glucan/%	HC0	HC0.5	HC2.5
Rhamnan	1.10 ± 0.04	1.18 ± 0.01	1.12 ± 0.04
Arabinan	0.34 ± 0.02	0.31 ± 0.03	0.37 ± 0.03
Xylan	4.43 ± 0.15	4.69 ± 0.04	5.16 ± 0.04
Mannan	1.36 ± 0.06	1.33 ± 0.02	1.36 ± 0.08
Galactan	2.04 ± 0.06	2.07 ± 0.03	1.99 ± 0.01
Yield of neutral carbohydrate */%	85.0 ± 0.3	85.2 ± 0.3	86.7 ± 0.3

* Including glucan yield.

**Table 6:** Cooking conditions of *Usu-mino-gami* *.

Sample	Production Year	Cooking Chemical	Alkaline Concentration/%	Cooking time/h	Duration before Washing/h	Gramarge *^4^ /g m^−2^	Thickness *^4^ /μm
S2 *^1^	2012	Na_2_CO_3_	10, 40	1(10%) + 1/6(40%)	12	15.9 ± 0.6	55.3 ± 4
N2 *^1^			13	2		14.8 ± 0.9	51.1 ± 4
N2(F) *^1^						17.7 ± 1.1	62.9 ± 4
W2 *^1^			10	1		16.7 ± 0.6	57.7 ± 3
Fu *^2^	Before 1994	Na_2_CO_3_	(12–13) *^3^	(1) *^3^	(2–4) *^3^	9.8 ± 0.8	48 ± 4

* This table is copied from a previous paper [1]. *^1^ Samples were prepared by Mr. Satoshi Hasegawa. N2(F) was prepared from washed pulp (N2) in a cloth bag (*Fukuroarai*) with water before sheet preparation. *^2^ Fu was prepared by Mr. Kozo Furuta, one of the best *usu-mino-gami* craftsmen ever. *^3^ According to the survey by Yagihashi [16]. *^4^ Mean ± standard deviation (N = 10).

**Table 7:** Cooking conditions of *kozo* sheet *.

Sample	Cooking Chemical	Alkaline Concentration/%	Cooking Time/h	Duration before Washing/h	Gramarge *^1^ /g m^−2^	Thickness *^1^ /μm
KC1 (KC1-10)	Na_2_CO_3_	10	1	1	26.8 ± 0.9	64 ± 3
KC1.5			1.5		29.7 ± 1.4	64 ± 6
KC2			2		29.5 ± 1.6	68 ± 5
KC2.5			2.5		34.8 ± 1.8	66 ± 4
KC3.5			3.5		33.2 ± 2.1	68 ± 4
KC1-12		12	1		28.0 ± 1.5	52 ± 2
KC1-14		14	1		27.1 ± 1.4	53 ± 1
KC1-16		16	1		26.6 ± 1.2	53 ± 2
KH0.5	NaOH	10	0.5		26.5 ± 1.9	59 ± 5
KH1			1		25.8 ± 1.5	62 ± 3
KH1.5			1.5		31.8 ± 2.4	65 ± 3
KH2			2		29.1 ± 2.0	58 ± 4
KH2.5			2.5		28.9 ± 2.6	60 ± 5

* This table is copied from a previous paper [2]. *^1^ Mean ± standard deviation (N = 10).

**Table 8:** Addition of glucuronoxylan to *kozo* pulp.

Sample	Cooking Chemical	Alkaline Concentration/%	Cooking Time/h	Amount of Glucuronoxylan Addition/g
HC0	Na_2_CO_3_	10	1	0
HC0.5				0.5
HC2.5				2.5

## Data Availability

Not applicable.
